# Correction: Self-collected and clinician-collected anal swabs show modest agreement for HPV genotyping

**DOI:** 10.1371/journal.pone.0288418

**Published:** 2023-07-07

**Authors:** Racheal S. Dube Mandishora, Trine B. Rounge, Megan Fitzpatrick, Irene Kraus Christiansen, Ole Herman Ambur, Sonja Lagström, Babill Stray-Pedersen, Massimo Tommasino, Joel Palefsky, Zvavahera M. Chirenje

There are errors in the Acknowledgments section. The correct Acknowledgments are as follows:

We would like to acknowledge Maria Da Costa (UCSF), who consistently gave counsel during sample collection and DNA extraction, Vasco Chikwasha for his statistical expertise, which he contributed during the proposal writing stages and Eugénie Lohmann for her contribution with R-studio commands. We also express our gratitude to Sister Mucheche, the nurse who recruited the women, the personnel at UZCHS-CTRC laboratory, the Norwegian HPV reference laboratory at Akershus University Hospital for their molecular expertise and technical support and the Parirenyatwa Hospital VIAC clinic staff for assisting during the recruitment phase. We are also very grateful to Prof. Jane R. Montealegre and all the other reviewers for their constructive critique and suggestions.

Where authors are identified as personnel of the International Agency for Research on Cancer/World Health Organization, the authors alone are responsible for the views expressed in this article and they do not necessarily represent the decisions, policy, or views of the International Agency for Research on Cancer / World Health Organization.

The authors would like to dedicate this paper to Babill Stray-Pedersen, in fond memory of her tireless dedication to female reproductive health. Babill passed away before the submission of the final version of this manuscript. Corresponding author RSDM accepts responsibility for the integrity and validity of the data collected and analyzed.
