# Supplementary material for: Self-collected and clinician-collected anal swabs show modest agreement for HPV genotyping
Source: PLoS One. 2021 Apr 26;16(4):e0250426. doi: 10.1371/journal.pone.0250426 (PMC8075200; doi:10.1371/journal.pone.0250426)
Supplement: S1 File — (PDF) [file pone.0250426.s001.pdf]

DATA COLLECTION SHEET

Title: Molecular characterization and diversity of Human papilloma virus in Women from Zimbabwe.

Investigator: Racheal S. Dube-Mandishora

Participant Number ☐

(Nhamba dzenyu dzemuchirongwa ichii)

MEDICAL RESEARCH COUNCIL OF ZIMBABWE
2015 -01- 14
APPROVED
P.O. BOX CY 573 CAUSEWAY, HARARE

1. Age ☐

(Munemakore mangani?)

2. Parity ☐

(Mune vana vangani?)

3. Level of education (Makadzidza kusvika pachinhanho chipi)

Grade 7 (Giredi sevheni) ☐ O' level (Fomu four) ☐ A' level (Fomu six) ☐

Tertiary (kukoleji kana university) ☐

4. Residence (Munogara kupi) Urban (Mudhorobha) ☐ Rural (Kumusha) ☐

5. Have you ever had a pap smear done? Yes (Hongu) ☐ No (Kwete) ☐ Never heard of it (Handiyizivi) ☐

Makambotorwa pap smear here?

6. Sexual debut (Makatanga kusangana nemurume pabonde munemakore mangani) ☐

I prefer not to answer this question (Ndinosarudza kusapindura muvhunzo uyu) ☐

7. a) Do you use herbs or any traditional medicine? ☐

(munoshandisa here mishonga yechivanhu)

b) If yes, are they for the vagina? ☐

(Kana mati hongu, mushonga wacho ndewe panhengo dzechibereko here)

8. How many sexual acts have you had in the past month? ☐

(pamwedzi yapfuura masangana nemurume pabonde kangani)

I prefer not to answer this question (ndinosarudza kusapindura muvhunzo uyu) ☐

9. Marital status (Makaroworwa here)

Married (ndakaroworwa) ☐ Divorced (ndakasiyana nemurume) ☐

Co-habiting (handina kuroworwa asi ndinemurume wandinogara naye) ☐

10. Does your spouse/partner have other sexual partners that you know of? ☐

(Murume wenyu anevamwe vakadzi vaanosangana navo pabonde vamunoziva here)

I prefer not to answer this question (ndinosarudza kusapindura muvhunzo uyu) ☐

11. Number of sexual partners ☐

(Kunze kwemurume wenyu, mune vamwe varume vangani vamunonasangana navo pabonde)

I prefer not to answer this question (ndinosarudza kusapindura muvhunzo uyu) ☐

12. a) Is your husband/partner circumsised? (Murume wenyu akachechewudzwa here) ☐

I do not know (Handizivi) ☐

I prefer not to answer this question (ndinosarudza kusapindura muvhunzo uyu) ☐

If on number 11 you gave a number greater than 0, please answer question 12(b)

b) How many of your sexual partners other than your husband/partner are circumcised? (Kunze kwemurume wenyu, vangani panevarume vamunosangana navo pabonde vakachechewudzwa?) ☐

I do not know (Handizivi) ☐

I prefer not to answer this question (ndinosarudza kusapindura muvhunzo uyu) ☐

13. History of STI treatment/genital warts (makamborapwa siki /zvirwere zvepabonde here)

Yes (Hongu) ☐ No (kwete) ☐

I prefer not to answer this question (ndinosarudza kusapindura muvhunzo uyu) ☐

14. Do you use any contraception? (Munoshandisa dziviriro here yekuti musabata pamuviri)

Yes (Hongu) ☐ No (Kwete) ☐ ☐

If Yes, tick the contraception that you use from the list below. (kana mati hongu, ndeipi dziviriro yekuti musabata pamuviri yamunoshandisa panedzanyorwa pasi)

Tablets (mapiritsi) ☐ Specify the tablets (anonzi chii mapiritsi acho) ....

Loop (Loop inopfekerwa muchibereko) ☐

Diaphragm (Dayafremu inopfekerwa muchibereko) ☐

Jadelle (Jadelle inopfekerwa paruwoko) ☐

Depo-provera (Depo yekubayiwa) ☐

Condom (kondomu) ☐

Other .... ☐

15. a) Have you ever been tested for HIV? (Makambowongororwa wutachiwona hwe HIV here)

Yes (Hongu) ☐ Date of diagnosis (makawongororwa rini)....../...../......

No (Kwete) ☐

b) If Yes, what was your result? (Makapiwa mhinduro ipi pamakawongororwa)

Positive (ndinewutachiwona) ☐ Negative (Handina wutachiwona) ☐

16. Was CD4 Count done at diagnosis of HIV? (Pamakanzi munewutachiwona weHIV makaitwa CD4 here) Yes (Hongu) ☐ No (Kwete) ☐

If yes, what was the CD4 count (Kana mati hongu CD4 yanga iri ngani) ☐

17. a) Are you on HAART (munomwa mishonga inorwisa hutachiwona hwe HIV here)

Yes ☐ No ☐

b) Duration of HAART (mavanenguva yakareba sei muchimwa mishonga iyi)

☐ months (mwedzi) ☐ years(makore)

MEDICAL RESEARCH COUNCIL OF ZIMBABWE
2015 -01- 14
APPROVED
P.O. BOX CY 573 CAUSEWAY, HARARE
