# Supplementary material for: Self-collected and clinician-collected anal swabs show modest agreement for HPV genotyping
Source: PLoS One. 2021 Apr 26;16(4):e0250426. doi: 10.1371/journal.pone.0250426 (PMC8075200; doi:10.1371/journal.pone.0250426)
Supplement: S1 Table — (DOCX) [file pone.0250426.s002.docx]

**S1 Table. Comparison of sequence read counts for HPV 52 and HPV 62 in both CCAS and SCAS.**

| **Patient ID** | **HPV genotype** | **CCAS counts** | **SCAS counts** | **Change in read counts from CCAS to SCAS** |
| --- | --- | --- | --- | --- |
| 005 | 52 | 2986 | 647 | Decreased |
| 019 | 52 | 309 | 154 | Decreased |
| 020 | 52 | 630 | 1924 | Increased |
| 023 | 52 | 54637 | 12737 | Decreased |
| 027 | 52 | 44978 | 2102 | Decreased |
| 031 | 52 | 17525 | 1169 | Decreased |
| 034 | 52 | 33513 | 1 | Decreased |
| 055 | 52 | 6226 | 9485 | Increased |
| 164 | 52 | 12124 | 24957 | Increased |
| 005 | 62 | 15949 | 702 | Decreased |
| 030 | 62 | 20351 | 19327 | Decreased |
| 042 | 62 | 13026 | 267 | Decreased |
| 058 | 62 | 55668 | 24885 | Decreased |
| 068 | 62 | 38315 | 13063 | Decreased |
| 128 | 62 | 63 | 1149 | Increased |
| 136 | 62 | 7277 | 3724 | Decreased |
| 164 | 62 | 1602 | 4791 | Increased |
| 206 | 62 | 1370 | 4664 | Increased |
| 230 | 62 | 40 | 7004 | Increased |
| 250 | 62 | 4 | 6859 | Increased |
