# Supplementary material for: Self-collected and clinician-collected anal swabs show modest agreement for HPV genotyping
Source: PLoS One. 2021 Apr 26;16(4):e0250426. doi: 10.1371/journal.pone.0250426 (PMC8075200; doi:10.1371/journal.pone.0250426)
Supplement: Attachment — Submitted filename: v2Response to reviewers Plos.docx [file pone.0250426.s003.docx]

Dear Dr Michael Scheurer

We would like to thank you and the reviewers for the thorough assessment of our work and for the constructive feedback. In the following, we respond to all concerns and comments point-by-point.

**Editor comments**

**Journal Requirements**

When submitting your revision, we need you to address these additional requirements.

**Editor point 1:** Please ensure that your manuscript meets PLOS ONE's style requirements, including those for file naming. The PLOS ONE style templates can be found at

<https://journals.plos.org/plosone/s/file?id=wjVg/PLOSOne_formatting_sample_main_body.pdf> and

<https://journals.plos.org/plosone/s/file?id=ba62/PLOSOne_formatting_sample_title_authors_affiliations.pdf>

**Reply:** We have now formatted and renamed our documents to ensure that they meet the journal’s style.

**Editor point 2:** Please include additional information regarding the survey or questionnaire used in the study and ensure that you have provided sufficient details that others could replicate the analyses. For instance, if you developed a questionnaire as part of this study and it is not under a copyright more restrictive than CC-BY, please include a copy, in both the original language and English, as Supporting Information.

**Reply:** The questionnaire used in our study, written in both English and Shona, is now included as supplementary document 1. We have also indicated the inclusion of the document under section 2.1 L127-128.

**Editor point 3:** In your Methods section, please provide additional information about the participant recruitment method and the demographic details of your participants. Please ensure you have provided sufficient details to replicate the analyses such as:  a) a description of any inclusion/exclusion criteria that were applied to participant recruitment. b) a statement as to whether your sample can be considered representative of a larger population, c) a description of how participants were recruited, and d) descriptions of where participants were recruited and where the research took place.

**Reply:** a-d) A line that briefly states that sexually active women aged at least 18 years old were reporting for routine cervical cancer screening were included is now captured in section 2.2 L145-150, whilst L124-129 inform the reader of how and where the study was carried out and to what extent the sample set was representative of a bigger population.

**Editor point 4:** Please provide a sample size and power calculation in the Methods, or discuss the reasons for not performing one before study initiation.

**Reply:** The sample size calculation is now detailed in section 2.1 L130-138. The formular accounted for a power of 0.80.

**Editor point 5:** To comply with PLOS ONE submission guidelines, in your Methods section, please provide additional information regarding your statistical analyses, including the threshold set for statistical significance for the analyses. For more information on PLOS ONE's expectations for statistical reporting, please see <https://journals.plos.org/plosone/s/submission-guidelines.#loc-statistical-reporting>.

**Reply:** The authors have a working R-script that can be made available upon the readers’ requests directly from the corresponding author. However a summary of the analyses performed on R-Studio is now included on section 2.2.2 L192-213.

**Editor point 6:** In the Methods section, please provide the specific sequences of the primers used in the next-generation sequencing analysis.

**Reply:** We have now included specific sequences of the primers as Table 1. L188

[Note: HTML markup is below. Please do not edit.]

**Reviewer #1 comments**

**Reviewer #1:** This is an interesting manuscript describing concordance between self- and clinician-collected anal swabs for HPV DNA testing. Given increasing incidence of anal cancer worldwide and new guidelines for anal HPV screening, self-sampling may help screening programs achieve greater coverage. Thus, there is a need for research regarding utility of self-sampling vis a vis clinician sampling. I have the following comments and suggestions:

**Abstract**

**Reviewer #1:** I suggest changing the last sentence to, “Our data suggest that self-collected anal swabs can be used as an alternative to clinician-collected anal swabs for HPV DNA testing in a clinical setting.”

**Reply:** We have now incorporated the suggested wording on L61-63

**Introduction**

**Reviewer #1:** Paragraph 1 would benefit from mention of burden of disease. I suggest including data on burden of cervical cancer, narrowing in on burden is sub-Saharan Africa.

**Reply:** We have now included facts on the burden of disease in Low-middle income countries and in Zimbabwe. This is captured on L75-78.

**Reviewer #1:** Paragraph 2 “Although anal cancers are relatively rare, data from USA reports a gradual increase in the incidence of anal cancers especially in HIV-infected individuals, regardless of the antiretroviral (ART) era [5]. Furthermore, a cumulative incidence of 0.1% by age 75 years was reported in HIV uninfected individuals as compared to 1.5% in HIV infected individuals.

The increase in anal cancer seems to be broader than among HIV-infected individuals. I suggest revising to: Although anal cancers are relatively rare, data from USA indicates a significant increase in the incidence of anal cancers [REF: <https://doi.org/10.1093/jnci/djz219>]. The increase is particularly high among HIV-infected individuals in the anti-retroviral era[include data on increasing incidence].

Side note: “The increase is particularly high among HIV-infected individuals, regardless of the antiretroviral (ART) era [5].” I’m not sure that it’s “regardless” of the anti-retroviral era, rather than “in” the antiretroviral era. Isn’t the extension of life due to ART driving increasing cancer incidence among HIV+ individuals?

**Reply:** We are grateful for the suggestions and we have now incorporated them on L84-86. We have also cited the paper by Deshmukh et al. Regarding the side note, we used ‘regardless of the ART era’ to indicate that in as much as ART is meant to improve the immunological profiles of an individual, we still have consistent HPV infections leading to cancer. The reviewer points out a different school of thought that describes the extension of life, due to ART, leading to cancer development. Both points are now integrated and captured in L85-88.

**Reviewer #1:** Paragraph 1 and 2 jump around quite a bit. Also, since the paper doesn’t seek to fill the gap in data regarding burden of anal cancer in Zimbabwe, I don’t think it makes sense to set this up as an argument in the Intro. How about ending paragraph 1 with “Women with a history of cervical cancer, especially those with HPV/HIV co-infection, are at increased risk of developing anal cancers [4]. Screening for anal cancer is thus recommended for women with cervical lesions and/or who are HIV infected [9, 10].” And starting paragraph 2 with “Although anal cancers are relatively rare, data from USA indicates a significant increase in the incidence of anal cancers. The increase is particularly high among HIV-infected individuals in the anti-retroviral era. Zimbabwe has an HIV prevalence of almost 13% and cervical cancer contributes a third of all cancers among black Zimbabwean women. In 2016, the Zimbabwe Cancer Registry reported a gradual increase in anal cancers recorded in the past decade. Despite the increased prevalence of risk factors in the population and evidence of increasing incidence, anal cancer screening guidelines are not routinely followed in Zimbabwe.”

**Reply:** Paragraph 1 now ends with the sentence on screening for anal cancer and paragraph 2 begins with the incidence of anal cancer in USA. L82-86.

**Reviewer #1:** Given focus of paper on HPV genotypes and multiple infections, it would be helpful to reader to understand why different genotypes matter. Could this be woven into the intro? This is mentioned in Discussion, but I’d move to Intro

**Reply:** We agree with the reviewer, for the reader to grasp the importance of reporting HPV genotypes right from the beginning, we have now moved the lines from discussion to the last paragraph of the introduction. L112-119.

**Reviewer #1:** Paragraph 3 jumps between accuracy and acceptability and between anal and cervical swabs. I suggest walking the reader through the arguments to better set up the rationale for the study. Eg. “Utility of self-sample cervical swabs for HPV DNA testing has been demonstrated. Furthermore, self-collection of cervical swabs has shown to be highly acceptable. For anal screening, there is evidence that self-sampling is acceptable and patients tend to be more comfortable and less embarrassed when an anal swab is self-collected. Its clinical utility has been demonstrated for non-HPV studies [sidenote: which tests?] and for cytology. However, here is still scarce data with regards to self-collected sample for HPV DNA testing.”

**Reply:** With the reviewer 1’s suggested phrasing, we have now re-written paragraph 3 and improved on the general flow. L95-100.

**Methods**

**Reviewer #1:** Laboratory Methods—DNA extraction. Is this per manufacturers’ instruction? If so, I ‘m not sure that this level of detail is needed.

**Reply:** Yes, the extraction was as per manufacturer’s instruction. The extra detail on DNA extraction has now been deleted L164-166

**Results**

**Reviewer #1:** The results and (discussion) seem to jump back and forth between describing HPV genotypes and presence of HPV DNA. As written, I can’t follow where kappa statistic is for presence of HPV DNA or for specific genotypes.

**Reply:** To avoid losing the reader and to improve on the flow we have now used consistent terminology, “HPV genotypes” in all sections. The Kappa test is for presence of any HPV genotype.

**Discussion**

**Reviewer #1:** Postulate 3 seems very unlikely.

**Reply:** We have kept postulate 3 in the text and added the word “unlikely” to guide the readers. L332

**Reviewer #1:** Paragraphs 7 and 8 (lines 338-353) seem to go beyond the data and focus of the paper. Given this, and that Discussion gets quite lengthy, I’d suggest omitting these paragraphs.

**Reply:** Paragraphs 7 describes the distribution of HPV genotypes by HIV status and paragraph 8 briefly describes cervical disease (as detected by Visual-inspection-with-acetic acid). Inasmuch as these two paragraphs mainly discuss secondary data, they are important for the reader to have a full perspective of the sample population. Given that women who have a history of cervical lesions and/or HIV co-infection are at increased risk of anal HPV infection. We have re-written the beginning of paragraph 8, L342, to capture the relevance of the paragraphs.

**Reviewer #1:** Conclusion: “Cautiously recommend…” should be replaced with “Our data suggest that…” (see comments on abstract).

**Reply:** We agree with the reviewer, therefore the conclusion is now phrased as ‘our data suggest that….’ L61 and L375.

**Reviewer #2 comments**

**Reviewer #2:** The authors of this generally well-written manuscript were interested in assessing potential anal cancer screening methods that focus on exfoliated cell collection from the anal canal. They compared clinician-collected vs self-collected anal swabs (CCAS vs SCAS) from women attending a cervical cancer screening program in Zimbabwe. The analysis found higher HPV prevalence in SCAS but a higher number of genotypes in CCAS. The method for HPV detection was next generation sequencing and this method leads to a number of questions about the results. At times, the attention to differing options for cut-off read numbers (100 vs 500 vs 1000) obscures the HPV results and calls into question the method of detecting HPV. And if the paper doesn’t have a sound method for detecting HPV, then the HPV results that depend on that detection method, e.g., comparisons of CCAS and SCAS, are called into question. Overall recommendation is to decide which cut-off level is best, defend that decision in the methods with literature, and then report results only for that cut-off level. Until that cut-off level is understood to be clearly the right one, then recommendations for anal cancer screening based on these data may be inappropriate. In addition, since anal cancer screening methods is the reason this study was done, the analysis needs to pay more attention to reporting results of high-risk HPV and results within HIV-positive people.

**Abstract**

**Reviewer #2:** L53 – focus these results on high-risk types and difference by HIV status.

**Reply:** We summarise the detection of high-risk HPV genotypes in the discussion section, L302-311 and L348-351. The primary aim and scope of this manuscript was to determine whether the same HPV genotypes were detected in self-collected and clinician-collected swabs, therefore we reported all the genotypes, regardless of whether they are high risk or low-risk. In addition, the most detected genotypes did not include a large number of high-risk genotypes. This is probably because these were mostly ‘disease free’ women reporting for routine screening, a good sample population for the purposes of primarily comparing CCAS to SCAS. We agree that in future studies, where we will focus on HPV genotype distribution other than comparison of collection methods, we can explore in-depth the anal high-risk HPV genotypes and HIV status.

**Methods**

**Reviewer #2:** L134 – why were Dacron swabs chosen over other options like flocked swab?

**Reply:** Our choice was based on literature that reported dacron and flocked swabs having harvested similar amounts of cells. Although in some scenarios flocked swabs are deemed superior, the difference with dacron swabs is minimal. <https://www.ncbi.nlm.nih.gov/pmc/articles/PMC5717820/> and <https://pubmed.ncbi.nlm.nih.gov/21791907/>. In our setting dacron swabs are the most readily available synthetic swabs, therefore given their proven use, we chose them to avoid inconsistency. The likelihood of running out of flocked swabs and having to wait for very long periods for new procurements would have been high. The citations used for justifying the choice of dacron swabs are now included on L145.

**Reviewer #2:** L150 – who did the breaking of the swab into the cryovial? Participant or staff?

**Reply:** The collection procedure was explained to all participants by the recruiting nurse. Self-collected swabs were broken into the tube by the participant, immediately after swabbing. Clinician-collected swabs were broken into the tube by the clinician as soon as they completed the swabbing. This is now indicated under section 2.2 L155.

**Reviewer #2:** L177 – why is 500 better than 100 or 1000? It seems to me that the results can have very different interpretations if, say 100, is chosen instead of 500. There is speculation about false-positive and false-negative results in the discussion, but it seems this should be clearly laid out by the authors in the methods.

**Reply:** We agree with the reviewer that presenting more than 1 cut-off can be confusing to the readers, thus we have carefully chosen to report all our findings based on a 500 read pairs cut-off. Our choice is based on the analyses of our read pairs which had a mean of 26232 for CCAS and 19110 for SCAS. Due to the difference in means, instead of normalising we calculated for a slightly higher cut-off. Assuming limit of detection of 5%, our 500 cut-off allows us to filter any false positive results and aligns with clinical recommendations of 300-500 published by Petrackova and colleagues https://pubmed.ncbi.nlm.nih.gov/31552176/. Although, there are a few papers that suggest varied cut-offs, Meisal et al DOI:10.1371/journal.pone.0169074 and Cullen et al doi: [10.1016/j.pvr.2015.05.004](https://dx.doi.org/10.1016%2Fj.pvr.2015.05.004) , there is generally no consensus on the minimum required read counts using deep targeted sequencing therefore each laboratory sets its own parameters based on the depth of coverage, the average read pairs and the statistical analyses methods employed. We have now clearly presented our justification (L199-202) and consistently only reported results using a cut-off that’s at least 500 read pairs. Fortunately, one of the co-authors is now preparing a methods paper to suggest the best cut-offs for our laboratory.

**Reviewer #2:** L180 – it would be nice to see simple agreement in addition to kappa (agreement beyond chance).

**Reply:** We present both the Cohen’s Kappa and the McNemer because they complement each other. Kappa assumes the two raters are equally competent whilst McNemar allows for unequal raters. We agree that the Kappa assumes the observed agreement to be due to chance. Therefore we included a McNemar test to enhance the agreement analysis and to ensure a more valid interpretation, L210. The McNemar assumes that HPV genotypes detected from self-collected swabs are equal to those detected by clinician-collected swabs. To the best of our knowledge, the complementary effect of these two tests is sufficient to describe the agreement of the collection methods <https://pubmed.ncbi.nlm.nih.gov/18188809/> and https://pubmed.ncbi.nlm.nih.gov/10520333/.

**Reviewer #2:** L181 – 183 – this sentence is difficult to understand.

**Reply:** The sentence has now been rephrased**.** We have now specifically described how the plots were made using the HPV genotype frequencies. L201-203.

**Reviewer #2:** L_ - most assays assess whether or not a sample is adequate for testing. How is this assessed with NGS?

**Reply:** NGS detects betaglobin as part of a control step to check if the DNA is adequate for testing. However, our samples were treated differently because they had already undergone betaglobin testing as an upstream procedure for a different assay (dot-blot-hybridisation) in Zimbabwe, for a different research scope. This was not repeated before NGS. We have now indicated this in the methods section. L166-168

**Reviewer #2:** Is it possible that a participant could have waived the swab in the air and this swab was judged to be HPV-negative by the authors?

**Reply:** This is highly unlikely, because the participants had the procedure explained to them by the nurse, who also closely monitored any women who requested supervision. As soon as the swab was collected it was transferred into the cryotube.
 **Results**

**Reviewer #2:** Table 1 – what kind of contraception for the Yes group?

**Reply:** This question referred to any type of contraception. A follow-up question also captured the specific contraception; however, this detailed information is not relevant for the scope of this manuscript. Please see question 14 of the questionnaire, attached as supplementary document 1.

**Reviewer #2:** Does the multiple sexual partners variable mean the participant acknowledged having >1 concurrent partner at recruitment?

**Reply:** Yes, this is correct. The participants self-reported having more than 1 sexual partners at the time of recruitment. Please see the questionnaire attached as supplementary material on question numbers 9 to 11.

**Reviewer #2:** Supp table 2 I don’t understand the heading: “Number of positive CCAS and SCAS GENOTYPES with cut-off……” Do you mean, “Number of HPV positive CCAS and SCAS specimens with cut-off……”?

**Reply:** Thank you for this note, this was a typographic error. The caption for supplementary table 2 (which is now table 3) has now been corrected to “specimens” instead of “genotypes”. L226

**Reviewer #2:** Figure 1 A CCAS - HPV16 is typically the most prevalent genotype in other studies as it almost is for CCAS at the 100 cut-off. And it certainly is the most important type for anal cancer. Why not give preference to the most sensitive scenario for detecting HPV16? i.e., choose 100 as the cut-off. The authors state there may be too many false-positives; but the paper doesn’t seem to have a convincing argument that these HPV16 reads are mostly false-positive. Also, since you’re reporting data for three cut-offs, and this is not a methods paper about cut-offs, the message of this figure is that reasonable persons might believe that any one of these 3 cut-offs is closer to the truth.

**Reply:** We have considered your suggestions and settled for a cut-off of 500. This is based on the explanation given above, for your question on L177. Concerning HPV 16 prevalence, indeed there seems to be a reduction in the cases but it was not statistically significant. Furthermore, with NGS we expect the sensitivity to be standard for all genotypes. We are also of the view that presenting an optimum cut-off will be better regardless of the loss of seemingly ‘positive’ samples because if they do not meet the optimum cut-off, we consider them to be negative. We also agree that presenting more than one cut-off is not suited for the scope of this paper. These will now be presented in a different methods manuscript that some of the co-authors are working on. To avoid misleading the readers, we have removed Figure 1. This will be best suited for a methods manuscript.

**Reviewer #2:** Fig 2 A and B – the grid lines fall in odd intervals making it tough to assess the frequency for any given column. B – This figure makes no sense to me. It does not communicate the level of concordance in any way that I can understand.

**Reply:** The authors have taken a closer look at figure 2 (which is now renamed to Figure 1) and we agree that the way the frequencies were presented may have been confusing. We have now relabelled the y-axis to present frequencies as whole numbers as opposed to the 0.5 intervals. The main message conveyed by the figure is the difference of frequencies of the HPV genotypes detected by each method (A), whilst (B) further gives a pictorial illustration of instances when an HPV genotype was detected by either of the collection methods or by both.

**Reviewer #2:** L207 – “type distributions were similar regardless of …cutoffs”….this doesn’t seem to be true since HPV16 – the most important type for anal cancer - has a prevalence of ~13% in CCAS with 100 reads. Prevalence of HPV 16 is otherwise 5 or 6 percent. L209 – reason for choosing 500 over 100 cutoff is very briefly addressed but reason for choosing 500 over 1000 is not addressed. Again, it seems this should be addressed in the methods.

**Reply:** Similar to the response to Figure 1 above. We have now justified the use of 500 as a cut-off, therefore we have now discarded the need to compare HPV genotypes based on cut-offs, as we agree that this is not a methods paper.

**Reviewer #2:** L221 – overall positivity for each swabbing group is central to this paper and should appear in a table or figure. There are too many data in the results narrative that are not included in a table or figure. In this area I would also expect to see results that are specific to high-risk types since it’s a cancer outcome that the authors care about most.

**Reply:** Regarding the overall positivity for each swabbing group, we have now presented these in table 3 L229, previously labelled as supplementary table 1 and 2. We agree with the reviewer, cancer outcome is a critical end point which we wish to prevent by providing data that can offer more screening options. In the context of cancer development, high-risk genotypes have the most clinical relevance. However, the primary aim and scope of this manuscript was to determine whether the same HPV genotypes were detected in self-collected and clinician-collected swabs, therefore we reported all the genotypes including those with less clinical relevance. Having determined and described the agreement using a broad array of HPV genotypes, we envision that when applications are being made in a clinical setting, the clinician will receive a report specifying the clinically relevant HPV genotypes.

**Reviewer #2:** L227 – why calculate kappa for all genotypes when only a few are responsible for almost all anal cancer?

**Reply:** The reviewer has rightfully highlighted that high-risk HPV genotypes have the most clinical relevance. We used Illumina next-generation-sequencing which detects a broad array of HPV genotypes, thus our overall HPV anal tests should report good accuracy for all HPV genotypes. The primary aim and scope of this manuscript was to determine whether the same HPV genotypes were detected in self-collected and clinician-collected swabs, therefore we reported all the genotypes including those with less clinical relevance.

**Reviewer #2:** L252 – It would be nice to see these data in a table stratified by HIV status.

**Reply:** The scope of this manuscript is to compare two methods and we do not expect the HIV status of the women to affect the accuracy of the test, therefore we do not present the HPV genotype agreement of CCAS and SCAS by HIV status. However, we give a summary description of the HIV status of the sample population based on the gold-standard, which is the clinician-collected in this case. This gives the reader a good indication of the HPV/HIV co-infection in this group of women. Sections 3.3 L260-270.

**Discussion**

**Reviewer #2:** L275 – Other studies have also compared CCAS and SCAS for cytology – Chin-Hong, 2008 and Cranston, 2004

**Reply:** Thank you for highlighting these important studies to us. The line has now been rephrased and the citations are now updated L98 and L282-283.

**Reviewer #2:** L302 – Add citation for claim that HPV53 is possibly carcinogenic.

**Reply:** A citation (Meyer et al 2001) has now been added and the phrase is also slightly modified to “probable high-risk”. L309-310.

**Reviewer #2:** L312-313. If the observations are dependent as you suggest (which makes sense), then the kappa statistic is not appropriate since it requires independent observations. Add this to limitations.

**Reply:** We have now highlighted this limitation on L363-370.

**Reviewer #2:** L328 – do you mean 40% of women with HPV had multiple infections?

**Reply:** Yes, 40% of women with HPV had multiple infections. We have added the phrase “of HPV infected” to clearly capture this L335.

**Reviewer #2:** L360 – this range is troubling, because it implies that the McNemar test was almost significant at one of the cut-off thresholds (I presume 100), i.e., at one of the cut-off levels, the p-value was 0.06 for a difference between CCAS and SCAS.

**Reply:** Thank you for this note. We have chosen our best cut-off to be 500, as described in the earlier responses in this document. We have now removed the comparisons that may confuse the reader. We agree that at one of the cut-offs the p-value of 0.06 was boarder line significant, therefore it should be confirmed using a larger sample size in other future studies.

**Reviewer #2:** L361 – the authors indicate here that they have adjusted the methods of the study (i.e., chosen a preferred cut-off threshold) in order to maintain statistical significance in their analysis. This is not appropriate. It brings me back to my central concern: that the authors need to justify which cut-off is most appropriate with literature. And if that’s not possible, then maybe a methods paper (that does not make recommendations on anal cancer screening) is most appropriate for these data.

**Reply:** We agree that our inferences may have not been well spelt out. The comparison of cut-offs was not appropriate for this paper. We will publish such comparisons in a different paper. To avoid any misinterpretations by the reader we have now presented all our data based on the optimum cut-off of 500 as justified in the response to similar comments above.

**Reviewer #2:** L366. Other than limitation of funds, there is not a clear listing of limitations of this study. One of these might be that kappa assumes the two raters are equally competent which is not the case here.

**Reply:** We have now collated our limitations and presented them on L360-366 and L363-370. We present the Kappa and McNemer because they complement each other. Kappa assumes the two raters are equally competent whilst McNemar allows for unequal raters.
