# Supplementary material for: Self-collected and clinician-collected anal swabs show modest agreement for HPV genotyping
Source: PLoS One. 2021 Apr 26;16(4):e0250426. doi: 10.1371/journal.pone.0250426 (PMC8075200; doi:10.1371/journal.pone.0250426)
Supplement: Attachment — Submitted filename: V2_2nd Response to reviewers_100221.doc [file pone.0250426.s004.doc]

**REF: PONE-D-20-19224R1-SELF-COLLECTED AND CLINICIAN-COLLECTED ANAL SWABS SHOW MODEST AGREEMENT FOR HPV GENOTYPING**

Dear Dr Michael Scheurer

We would like to thank you and the reviewers for the additional comments on our work and for the constructive feedback. In the following, we respond to all concerns and comments point-by-point.

**Additional Editor Comments**

**Comment 1:** In addressing the remaining reviewers' comments below, please also ensure that you have cited the correct references to support the use of the statistical tests that you used.

**Response:** Additional references have now been included, such as Ranganathan P, Pramesh CS, Aggarwal R. Common pitfalls in statistical analysis: Measures of agreement. Perspect Clin Res. 2017 Oct-Dec;8(4):187-191.

**Comment 2:** Please confirm that your manuscript meets the PLOS One data submission/availability policy. In particular, the policy states that "data points used to create, for example, means and medians, need to be available."

**Response:** The authors have a working R-script that can be made available upon the readers’ requests directly from the corresponding author.

**Reviewer #1 Comments to the Author**

**Comment 1:** The authors have satisfactorily addressed all of my previous comments. However, I have some concerns that I had overlooked regarding the statistical analysis, specifically the use of McNemar’s test as a test of agreement (see below). The authors conclude that there is moderate agreement (based on kappa=0.55), but state that this was not statistically significant based on McNemar’s test. However, McNemar’s is a test of proportions, rather than agreement. Thus, the p-value used to support the conclusion is only able to conclude that the proportion of a particular genotype was not significantly different between CCAS and SCAS, rather than indicating agreement. My other comments relate to minor issues that should be easy to address.

**Response:** Thank you for highlighting the interpretation of these statistical analyses. To avoid confusion for the readers, the authors have now separated the two statistical analyses to represent (1) measure of agreement (L54-55) and (2) test of proportions (L55). This has also been applied throughout the manuscript in the methods (L223-225), results (L253-255) and discussion (L314-318). The tests are not used as one but rather as two separate complimentary tests.

**Comment 2:** Abstract: There’s a small typo in conclusions: “with no statistically-significant difference.”

**Response:**
We have now deleted the “none” and used “no” on L58.

**Introduction**

**Comment 3:** I appreciate the authors’ clarification of the increasing incidence of anal cancer among HIV-infected individuals in the era of ART: “The increase is particularly high among HIV-infected individuals regardless of a repaired immune system due to ART, and conversely, extension to life due to ART tends to give time for cancer to develop [9].” Just to clarify, is the null association at the individual level (HIV+ individuals on ART are as likely than those not on ART to develop anal cancer) or at the ecologic level (despite advances and coverage in ART, anal cancer incidence continues to rise among HIV+)? If the latter, I think it may be better to say something like, “The increase is particularly high among HIV-infected individual despite the availability and widespread (?)/ increasing (?) use of ART, which improves individuals’ immunogenic profile. Conversely, ART extends life among HIV infected individuals, giving time for cancer to develop.” Also, please define ART on first use 101-103: The last sentence of this paragraph is repeated from above: “Furthermore, patients tend to be more comfortable and less embarrassed when an anal swab is self-collected [21].

**Response:** Yes, the intention was to explain the latter scenario. The null association is at an ecological level. We have therefore edited the paragraph to include the reviewer’s useful suggestions, L86-88. The acronym ART has now been introduced in L87. The repeated sentence has now been deleted from line 108.

**Comment 4:** 112-13: please define SCAS and CCAS on first use

**Response:** The acronyms SCAS and CCAS are now introduced on L121, in addition to the definition that was provided in the abstract L40.

**Methods**

**Comment 5:** 144-148 The information in this paragraph seems redundant with the paragraph below. I suggest omitting this paragraph and integrating any new information provided into the paragraph below.
Agreement was tested using kappa and McNamar’s test. However, McNamar’s test compares overall proportions, not agreement (see: (see: Ranganathan P, Pramesh CS, Aggarwal R. Common pitfalls in statistical analysis: Measures of agreement. Perspect Clin Res. 2017 Oct-Dec;8(4):187-191.). I’m not confident that this test can be used to support the authors’ conclusions.

**Response:** We agree with the reviewer, stating that agreement was tested using kappa and McNemar can be misleading. To remove this confusion, we have now separated the two statistical analyses to represent (1) measure of agreement (L54-55) and (2) test of proportions (L55). This is also explained in the responses above. The authors believe that both statistical tests are useful. We are also conscious of the fact that there is no perfect test to evaluate agreement and it’s a classical source of debate. Both tests are commonly used together to complement each other https://pubmed.ncbi.nlm.nih.gov/10520333/. McNemar is indeed a test of proportion, but the proportion of discordant pairs (ie +/- vs -/+), which gives useful information when evaluating (dis)agreement based on proportions. Indeed, as mentioned by Ranganathan et al, a non-significant McNemar test is not enough to say the agreement is good. It’s however still useful, if interpreted correctly. We have now further clarified our interpretations and limitations in L399-405 of the Discussion section.

**Results**

**Comment 6:** Table 3 says CCAS: 124 HPV + samples, SCAS:114 HPV + samples. But paragraph below says 67 and 75, respectively. What is Table 3, last column referring to?

**Response:** We apologise for the misleading column. The 124 and 114 refer to every HPV positivity regardless of multiple infections. In other words, for CCAS, the total read pairs amounted to 124 HPV genotypes in 67 women. This is because some of the 67 women had multiple HPV genotypes, such that there were 32 different HPV genotypes repeatedly detected in 67 women. However, to avoid confusing the reader, the column is now titled “number of HPV positive women”.

**Comment 7:** 249-250: This sentence is confusing and I’m not sure of relevance: In addition, 13/29 of these women had multiple infections in both SCAS and CCAS regardless of HPV genotype and number of infections.

**Response:** We have now removed the statement.

**Comment 8:** Figure 3. I’m confused as to why there’s a single p-value for the McNamar’s test. McNamar’s is used for paired nominal data in a 2x2 contingency table. So, for each genotype CCAS vs. SCAS and genotype present versus genotype absent. It seems like there should be a test statistic and associated p-value for each genotype-comparison, rather than a global McNamar’t test statistic and p-value.

**Response:** The reviewer raises an important question which fortunately we also touched on in the first rebuttal. In our data, each genotype was considered independent because we were most interested in knowing how much HPV was detected in either methods or in comparison. Given the broad array of HPV genotypes that the Illumina sequencer could detect, it was complicated to break down our analyses into each and every HPV genotype. Discordant results were defined as those that were HPV positive on one specimen type while negative on the other. However, we have highlighted this under limitations L294-299 and L371-384. Furthermore, NGS is a highly sensitive assay (DOI:10.1038/s41598-018-36669-6 ) and we do not expect a difference in the capacity for detecting HPV genotypes assuming there are infected cells on the swabs, rather, our research question is whether each collection method harvests enough cells for HPV genotypes to be detected, when comparing two detection methods.

**Discussion**

**Comment 9:** 327-334: The same has been observed for self-collected versus clinician-collected cervical swabs [See Arbyn et al 2018-- <https://pubmed.ncbi.nlm.nih.gov/30518635/>], attributed to vaginal sampling with self-collection versus cervical sampling with CC.

**Response:** Thank you for this important reference. We have included it in our discussion, L340-342

**Comment 10:** The authors conclude that there is moderate agreement (based on kappa=0.55), but state that this was not statistically significant, based on McNamar’s test. However, McNamar’s isn’t a test of agreement. Thus, I’m not certain that the data support this conclusion.

**Response:** We agree with the reviewer. Yes, the McNemar is best referred to as a method to ‘evaluate agreement’, as opposed to a ‘test of agreement’. We have now carefully rephrased the abstract and conclusion to fully portray what both statistical tests do and mean. This has now been applied throughout the manuscript in the methods (L210-212), results (L238-240) and discussion (L314-318). We have also toned down our conclusion to ensure that the readers fully understand what our results mean L287-293.

**Reviewer #2 Comments to the Author**

**Comment 1:** Thanks for addressing my comments. I appreciate the authors complete and thoughtful replies. I would only point out that the following critique does seem to have been addressed in the revised manuscript.
L312-313. If the observations are dependent as you suggest (which makes sense), then the kappa statistic is not appropriate since it requires independent observations. Add this to limitations.
Reply: We have now highlighted this limitation on L363-370.

**Response:** Thank you for the comment, but unfortunately there is probably a mix up on the line numbering and we are not sure if we have fully understood your comment. Nonetheless, as mentioned above, the authors believe that both statistical tests are useful. We are also conscious of the fact that there is no perfect test to evaluate agreement. Both tests are commonly used together to complement each other. However, we have spelt out the limitations in L294-299 and L371-384. If the reviewer is talking about the different hypotheses for higher detection with SC, (because L302 corresponds to L232 on the original pdf and L328 to L259), then the fact that the second swab is influenced by the first one, is just a hypothesis, the observations (SC and CC) can be considered independent. We have reiterated our speculative hypotheses for these differences in L322-323.
