# Supplementary material for: Self-collected and clinician-collected anal swabs show modest agreement for HPV genotyping
Source: PLoS One. 2021 Apr 26;16(4):e0250426. doi: 10.1371/journal.pone.0250426 (PMC8075200; doi:10.1371/journal.pone.0250426)
Supplement: Attachment — Submitted filename: V1_3rd Response to reviewers.docx [file pone.0250426.s005.docx]

**REF: PONE-D-20-19224R2-SELF-COLLECTED AND CLINICIAN-COLLECTED ANAL SWABS SHOW MODEST AGREEMENT FOR HPV GENOTYPING.**

Dear Dr Michael Scheurer

We would like to thank you and the reviewers for the additional comments on our work and for the constructive feedback. In the following, we respond to all concerns and comments point-by-point.

**Review # 1 Comments to the Author**

**Comment 1**: The authors have fully addressed all of my comments and suggestions. I have a few minor suggestions related to how the kappa and McNemar’s are presented and a few minor edits in the Discussion.
Abstract (and throughout) Thank you for clarifying that kappa and McNamar’s were two separate tests used to evaluate concordance. For the sake of clarity, could the following edits be made to the abstract:
Methods: Currently: Level of agreement was calculated using the kappa and McNemar tests. Suggested: Level of agreement of HPV genotypes between CCAS and SCAS was calculated using the kappa statistic. McNemar tests were used to evaluate agreement in the proportion of genotypes detected by either method.
The same info is available elsewhere in the abstract, but it would improve clarity to include it in the Methods so that readers can better understand the Results.

**Response:** We have now included the suggested phrasing on L48-51.

**Comment 2:** Methods: Similarly, I’d clarify how the statistics were used in the last paragraph of the Methods. Currently: To evaluate agreement of the two collection methods, the McNemar non-parametric test was performed. A kappa test for agreement was also performed to complement the McNemar [38–40]. Suggested: A kappa test was used to evaluate agreement of HPV genotypes across the two collection methods. The McNemar’s non-parametric test was performed to evaluated agreement in the proportion of genotypes detected by either method.

**Response:** We have now included the suggested phrasing on L213-216.

**Comment 3:** Discussion, line 294-300: Current: Based on the moderate level of agreement of HPV genotypes between the two methods, signified by 0.55 in kappa value (k), our data suggest that researchers can use either methods for collection. Furthermore, to evaluate agreement of the methods, McNemar gave a Chi-square value of 0.75 (p=0.39), which showed that the difference in detection rates on the two methods was not statistically significant. Suggested: Based on the moderate level of agreement of HPV genotypes between the two methods, indicated by 0.55 in kappa value (k) and a non-significant test of proportions, our data suggest that researchers can use either methods for collection.

**Response:** We have now included the suggested phrasing on L294-297.

**Comment 4:** Line 351-353 (minor edit): Arbyn et al had similar observations, reporting HPV detection rates 2.28 times higher in self-collected versus clinician-collected vaginal swabs [45].

**Response:** We have now added the word “versus” to the sentence, L343-345.

**Comment 5:** Line 384-389, Current: Indeed, as mentioned by Ranganathan et al, a non-significant McNemar test is not always enough to say the agreement is good. It’s however still useful, if interpreted correctly [51]. In our case, the McNemar is indeed a test of proportion, but the proportion of discordant pairs (ie +/- vs -/+), which gave useful information for evaluating (dis)agreement of HPV genotype detection. Suggested: Indeed, as mentioned by Ranganathan et al, a non-significant McNemar test alone is not sufficient to indicate good agreement. However, it is still a useful statistic, if interpreted correctly [51]. In our case, the McNemar test indicated that the proportion of discordant pairs (ie +/- vs -/+) was not statistically significant, providing useful information for evaluating (dis)agreement of HPV genotype detection.

**Response:** We have now rephrased L388-393, to in cooperate the suggestion.

**Additional edits by authors:** Additional information on **author contributions** has now been included on L428-431, to indicate the unfortunate loss of one of our co-authors BSP, who passed away before the submission of the final version. We have also taken the opportunity to thank the reviewers for their contributions in remoulding this manuscript, L408 specifically mentions Prof. Jane Montealegre and those who also remained anonymous.

**Do you want your identity to be public for this peer review?** For information about this choice, including consent withdrawal, please see our [Privacy Policy](https://www.plos.org/privacy-policy).

Reviewer #1: **Yes:** Jane R Montealegre
